# Evidence of mixotrophic carbon-capture by n-butanol-producer *Clostridium beijerinckii*

**DOI:** 10.1038/s41598-017-12962-8

**Published:** 2017-10-06

**Authors:** W. J. Sandoval-Espinola, M. S. Chinn, M. R. Thon, J. M. Bruno-Bárcena

**Affiliations:** 1 Department of Plant and Microbial Biology, North Carolina State University, Raleigh, NC USA; 2 Department of Biological and Agricultural Engineering, North Carolina State University, Raleigh, NC USA; 3 Instituto Hispano-Luso de Investigaciones Agrarias (CIALE), Department of Microbiology and Genetics, University of Salamanca, Calle Del Duero 12, Villamayor, 37185 Spain; 4 Present Address: Department of Chemistry & Chemical Biology, Harvard University, 12 Oxford Street, Cambridge, MA 02138 USA

## Abstract

Recent efforts to combat increasing greenhouse gas emissions include their capture into advanced biofuels, such as butanol. Traditionally, biobutanol research has been centered solely on its generation from sugars. Our results show partial re-assimilation of CO_2_ and H_2_ by n-butanol-producer *C*. *beijerinckii*. This was detected as synchronous CO_2_/H_2_ oscillations by direct (real-time) monitoring of their fermentation gasses. Additional functional analysis demonstrated increased total carbon recovery above heterotrophic values associated to mixotrophic assimilation of synthesis gas (H_2_, CO_2_ and CO). This was further confirmed using ^13^C-Tracer experiments feeding ^13^CO_2_ and measuring the resulting labeled products. Genome- and transcriptome-wide analysis revealed transcription of key C-1 capture and additional energy conservation genes, including partial Wood-Ljungdahl and complete reversed pyruvate ferredoxin oxidoreductase / pyruvate-formate-lyase-dependent (rPFOR/Pfl) pathways. Therefore, this report provides direct genetic and physiological evidences of mixotrophic inorganic carbon-capture by *C*. *beijerinckii*.

## Introduction

Current societal efforts require solutions to address increasing greenhouse gas emissions^1^. Accordingly, carbon-capture and its biotransformation into useful value-added commodities, including biofuels, has become a growing area of research. Thanks to a better understanding of pathways such as the Wood-Ljungdahl (WL), and the recently described reversed-pyruvate ferredoxin oxidoreductase (rPFOR)/pyruvate-formate-lyase-dependent (Pfl) carbon assimilation^2^, more microorganisms can potentially be screened for presence of these pathways using physiological signals. These pathways allow the incorporation of one-carbon (C-1) molecules into Acetyl-CoA. This is a key molecule that can be subsequently bio-transformed into value-added molecules such as acetate or ethanol^3–7^. Currently, butanol is considered one of the ideal advanced renewable fuel due to a number of favorable properties and applications^8–10^. For example, it can be used unblended in unmodified car engines and is compatible with current oil infrastructure^11^. However, only recently the assimilation of synthesis gas (containing H_2_, CO and CO_2_) into butanol, by natural or genetically modified microbes, has been assessed, and remains in the early stages of development^5,6,12,13^. As a result, seeking to achieve cost-competitive butanol production, most research has focused on assessing the heterotrophic biotransformation of renewable feedstock by traditional solventogenic Clostridia. However, heterotrophic fermentations have the inherent limitation that 1/3 of carbon is lost in the form of CO_2_. Interestingly, the reported data shows significant variability in apparent final product yields, pointing towards overlooked metabolic capabilities^9,14–17^. With this in mind, we performed a deeper examination of the evolving fermentation gases as physiological signals, while assessing the assimilation of synthesis gas by the natural n-butanol producer *C*. *beijerinckii*.

## Results

### Real-time (in-line) fermentation gas monitoring reveals CO_2_ and H_2_ oscillations

**Figure 1:**
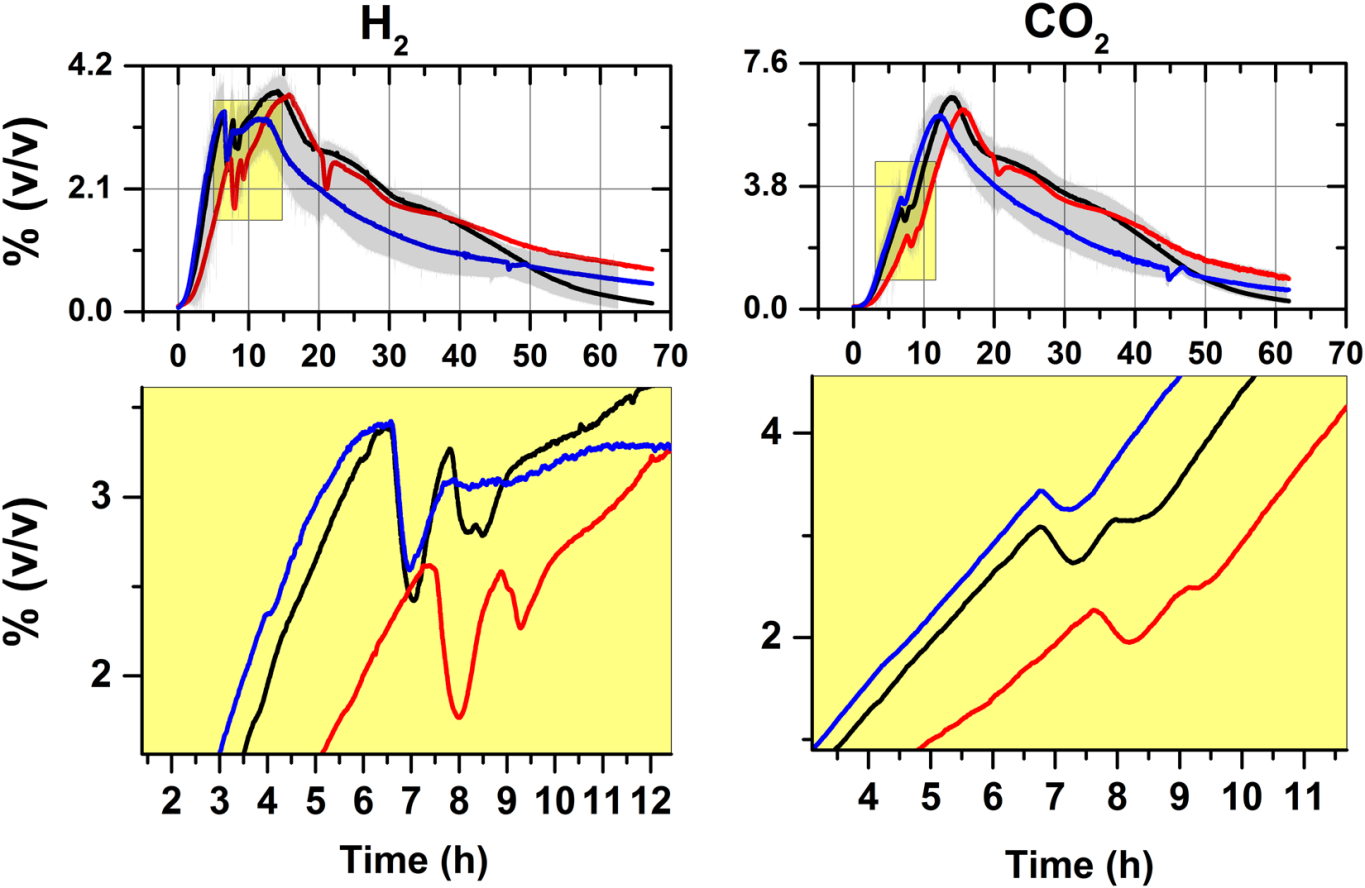
Direct monitoring of hydrogen and carbon dioxide evolution in the gas-phase during fed-batch fermentations by *C. beijerinckii*. H_2_ and CO_2_ evolution from three independent experiments performed in a Biostat B+ reactor using defined medium^20^ containing 6% (w/v) sucrose as limiting carbon and energy source with an initial and final volumes of 1000 mL and 1400 mL, respectively. Feeds (400 mL) contained 80 g sucrose fed at 0.08 mL/h, to reach a final concentration of 100 g/L (w/v) along with: Red line: only sugar was added; Black line: fresh whole medium and; Blue line: 2X trace components. Yellow boxes show detail of the H_2_ and CO_2_ oscillation. Fermentations were controlled at 250 rpm, 37 °C and pH 6.5 and constantly sparged (12.48 L/h) with nitrogen gas was achieved using mass flow controllers. Output gas-phase composition was continuously monitored and recorded using two analyzers: An EasyLine continuous analyzer, model EL3020 (ABB, Germany) and a Pfeiffer OmniStar quadrupole mass spectrometer.

We performed a series of fed-batch fermentations of *C*. *beijerinckii* while monitoring, in real-time (in-line), the evolving endogenous gasses. Interestingly, we observed in-phase, synchronous H_2_ and CO_2_ oscillations coinciding with late log-phase and the onset of solventogenesis (when H_2_ and CO_2_ reached ≈3% [v/v]) (Fig. 1). These types of fluctuations are normally observed in feedback-loop controls as a response to metabolic pathway changes^18,19^. Indeed, diauxic growth was evident from the decrease in the specific growth rate as H_2_ and CO_2_ resumed their accumulation (Fig. S1). Although we tested three feed compositions, (i.e. all while containing additional sucrose: (i) fresh complete medium; (ii) 2X trace components^20^; (iii) sucrose only) these had no effect on the gas oscillations. This suggested to us that neither organic carbon nor another medium component modulated this phenotype. Furthermore, the cells failed to utilize all the provided sugar, and its utilization varied significantly among experiments (Fig. S2, D, D1). In contrast, the kinetic and yield parameters for products and biomass did not vary significantly (Fig. S2–A1, B1, and C1; and Table S1). We used nitrogen (F = 12.48 L/h) as carrier/stripping for gas measurements. In batch (i.e. closed-system) without stripping CO_2_ and H_2_ accumulated in the head-space and reached 48 and 23%, respectively (not shown).

It was previously shown that recirculating endogenous H_2_ and CO_2_ during butanol fermentation (for maintaining anoxic conditions) allows for more sugar consumption and acid generation^14^. Additionally, increased product biosynthesis has been shown when electrochemical bioreactors were cultured with *C*. *acetobutylicum* in complex medium along with CO_2_^21^. Interestingly, the C-1 assimilation in bacteria is also a mechanism for redox balance, helping to sustain substrate uptake^22^. Nevertheless, C-1 assimilation has not been previously described in *Clostridium beijerinckii* ^6,7^.

### Genomic and indirect transcriptomic analysis indicates that *C*. *beijerinckii* has the genetic potential for C-1 assimilation

**Figure 2:**
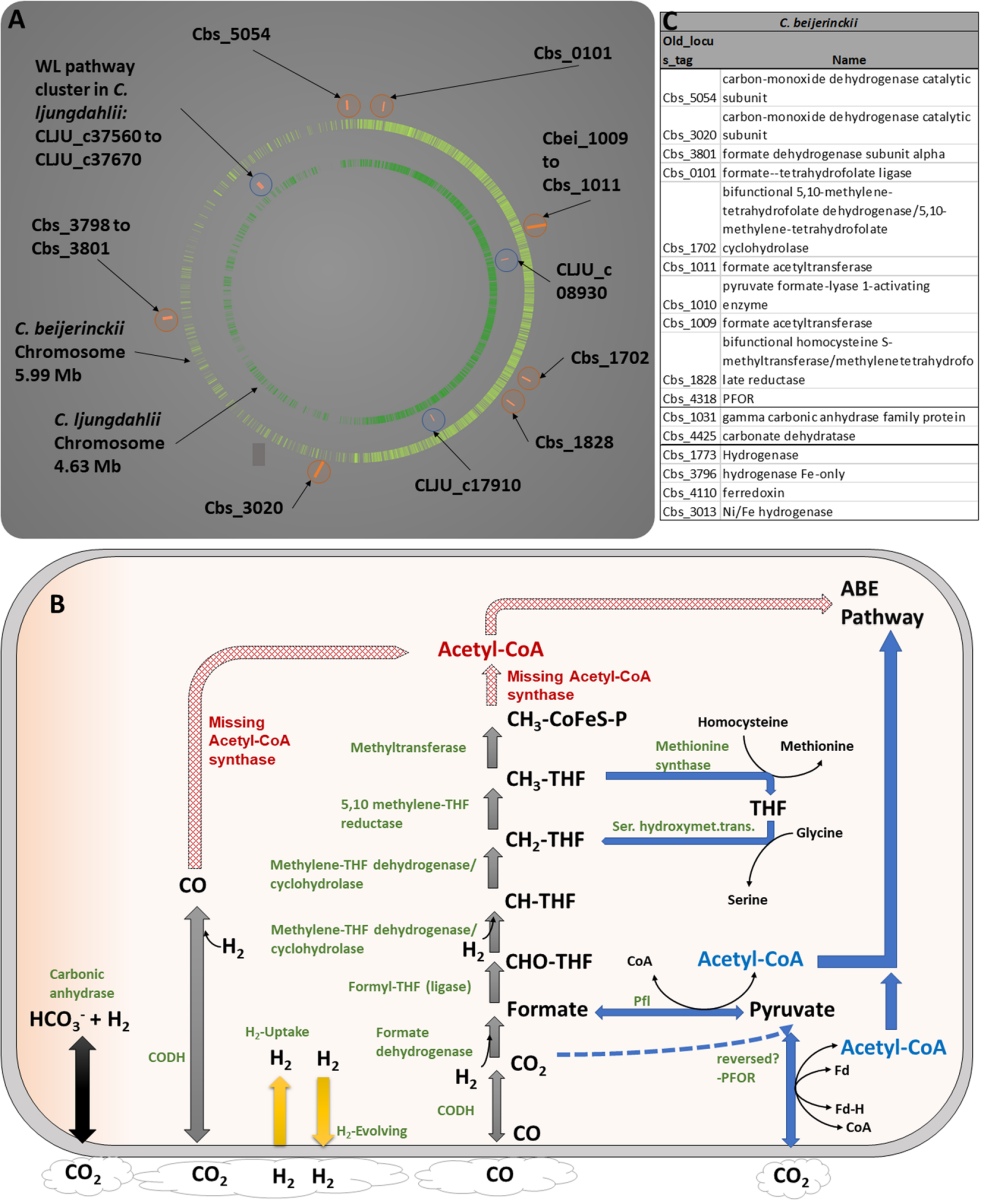
Partial Wood-Ljungdahl (WL) and reverse pyruvate: ferredoxin oxidoreductase/pyruvate-formate lyase (rPFOR/Pfl) pathways scheme in *C*. *beijerinckii* and chromosome localization of corresponding genes. (**A**) C-1 assimilation genes found in *C*. *beijerinckii* and *C*. *ljungdahlii* (WL pathway), mapped in their respective chromosomes. (**B**) Presumed partial WL and rPFOR/Pfl scheme pathways in *C*. *beijerinckii*. Red arrows indicate reactions predicted to be catalyzed by the CO dehydrogenase/Acetyl-CoA synthase complex, (acetyl-CoA is not coded in *C*. *beijerinckii* genome). Gray and blue arrows indicate reactions belonging to the methyl branch of the WL, and rPFOR/Pfl pathways, respectively. Black and yellow arrows indicate CO_2_ and H_2_ assimilation (and evolving) reactions. (**C**) Genes and their names associated to C-1 assimilation in *C*. *beijerinckii*.

To explain the gas oscillations and their potential assimilation, we explored the *C*. *beijerinckii* genome searching for genes related to C-1 assimilation, such as those associated to the WL or rPFOR/Pfl pathways^2,5,7,23^. We found open reading frames that putatively code for CO dehydrogenase (CODH) (Cbei_5054 and Cbei_3020), formate dehydrogenase and accessory genes (Cbei_3798 to Cbei_3801), formyl-THF ligase (Cbei_0101), methylene-THF dehydrogenase/cyclohydrolase (Cbei_1702) and methylene-THF reductase (Cbei_1828). The putative proteins encoded by these genes have high sequence identity to those of *Clostridium ljungdahlii*, the species most often utilized for ethanol generation from synthesis gas^5,24^ (Fig. 2A–B). However, as opposed to this species, *C*. *beijerinckii* does not contain annotated a gene coding specifically for an acetyl-CoA synthase, which is essential within the WL pathway. Furthermore, in *C*. *ljungdahlii*, most of the WL pathway genes are clustered, except a gene coding for a Ni-Fe-S containing CODH (CLJU_c17910), and a formate dehydrogenase (CLJU_c08930). The former is the main enzyme within the carbonyl branch of the WL pathway, and the final step of the methyl branch (or initial step, if CO is supplied). The latter initiates the methyl branch, allowing CO_2_ capture into formate. Together, they lead to the generation of acetyl-CoA via an acetyl-CoA synthase. Conversely, *C*. *beijerinckii* contains the homologous genes scattered through its chromosome (Fig. 2A). Interestingly, the annotated CODH and the formate dehydrogenase from *C*. *beijerinckii* have 77.62 and 72.23% sequence identity, respectively, to the corresponding genes of C. *ljungdahlii* localized outside its WL cluster. In addition to these genes, *C*. *beijerinckii* contains Fe-only and NiFe-hydrogenases (Cbei_1773, Cbei_3796, Cbei_4110 and Cbei_3013) with similarities to those of *C*. *ljungdahlii*. In this species, along with H_2_ generation, these enzymes have hydrogen uptake capabilities, providing extra reducing equivalents to its C-1-fixation pathway^5^.

*C*. *beijerinckii* also contains two putative Pfl-coding genes (Cbs_1009 and Cbs_1011), (both annotated as formate acetyltransferase, as is the case in *Clostridium thermocellum* [*pflB*, clo1313_1717)])^2^, and a putative pyruvate formate-lyase activating enzyme gene (Cbs_1010). The proteins coded by Cbs_1009 and Cbss_1011, and Cbs_1010, have ~63.5 and 44.4% sequence identity to those of *C*. *thermocellum*, respectively. This bacterium, while relying on a partial WL pathway (i.e. the methyl branch without the formate dehydrogenase), contains a reverse pyruvate ferredoxin oxidoreductase (clo1313_0673 and others) that combines acetyl-CoA and CO_2_ to generate pyruvate, which is then transformed into formate and acetyl-CoA by Pfl^2^. Interestingly, the *C*. *beijerinckii* pyruvate ferredoxin oxidoreductase (PFOR) (Cbs_4318) has 64.1% sequence identity to *C*. *thermocellum* rPFOR. The reverse reaction of PFOR has also been observed in other acetogenic and methanogenic bacteria, where this enzyme links the WL pathway and glycolysis^25^. Additional genes related to the rPFOR/Pfl pathway are a serine hydroxymethyltransferase and a methionine synthase, both of which are also encoded in *C*. *beijerinckii* chromosome (Cbs_1868, and Cbs_3100, Cbs_2329 and Cbs_1401, respectively).

**Figure 3:**
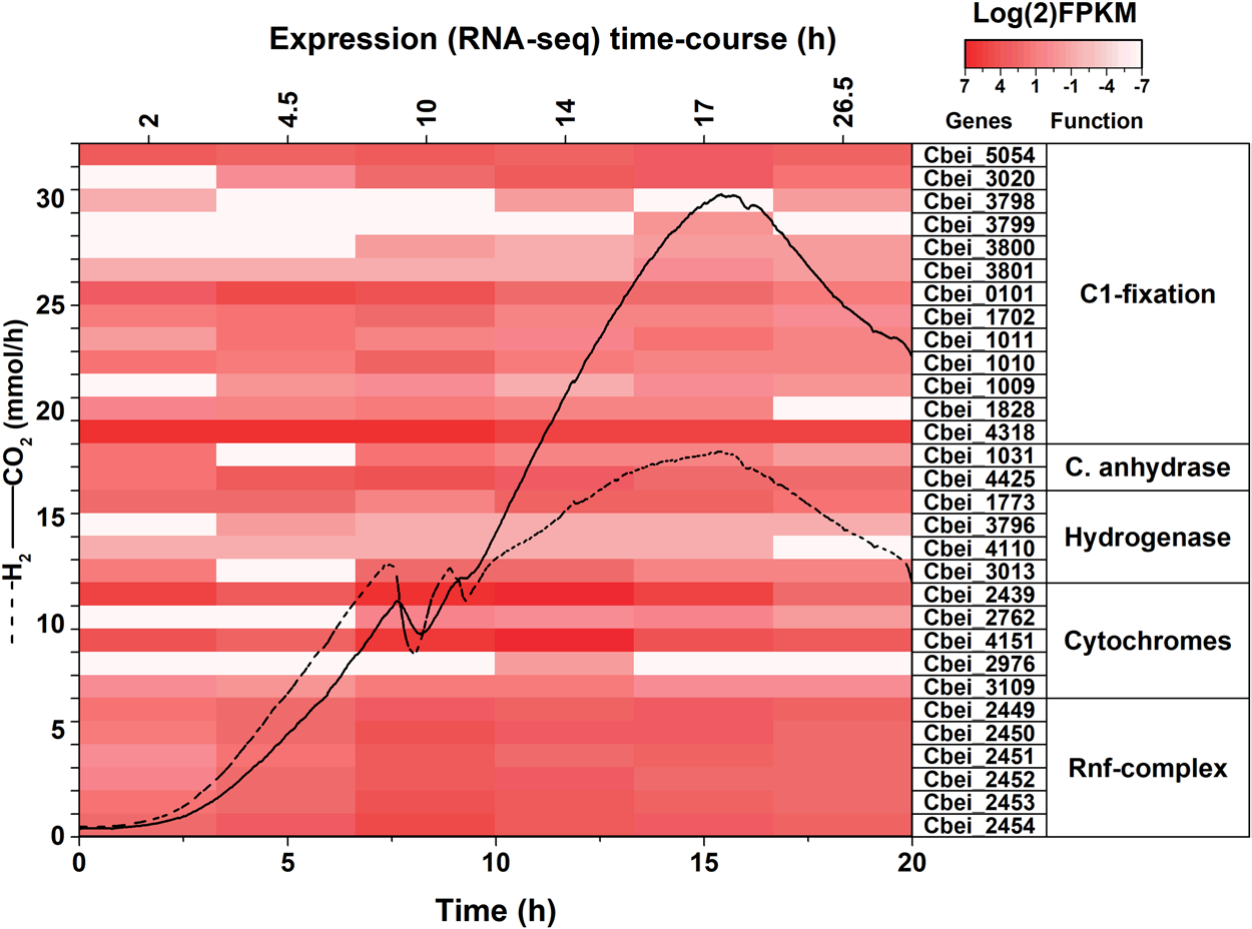
Time-course transcription profile of C-1 assimilation and energy conservation genes in *C*. *beijerinckii*. Time-course expression profiles of partial Wood-Ljungdahl and pyruvate: ferredoxin oxidoreductase/pyruvate-formate lyase (rPFOR/Pfl) predicted genes, along with genes related to energy conservation in *C*. *beijerinckii*. The FPKM (fragments per kilobase per million) were calculated from publicly available RNA-seq data^27^. Lines represent CO_2_ and H_2_ evolution in the gas-phase of *C*. *beijerinckii* growing in defined medium^20^, 37 °C, 250 rpm, and constantly sparged with pure nitrogen (12.48 L/h).

With these C-1 assimilation genes in focus, we performed an analysis of publicly available transcriptomic data from batch cultures of *C*. *beijerinckii* ^26^. An RNA-seq time-course experiment was previously reported by Wang *et al*.^27^ using cells growing in P2 medium sparged with pure nitrogen. After quality trimming and normalization for gene length and number of assembled reads, we found the putative genes required for C1-assimilation being expressed, either constitutively (Cbei_5054, Cbei_1828 and Cbei_4318) or differentially over time (Cbei_1702, Cbei_0101, Cbei_3801, Cbei_3794, Cbei_3798, Cbei_3799, Cbei_3800, Cbei_3020, Cbei_1010 and Cbei_1011) (Figs 3 and S3). After mapping this transcriptomic response to our gas oscillation data, we identified expression changes that coincided with this phenotype, indirectly pointing towards the re-assimilation of these gases (Figs 1 and 3). Among these genes, formate dehydrogenase and its accessory genes showed the lowest expression in the evaluated experimental condition (i.e. low biomass and CO_2_/H_2_ concentration, and nitrogen atmosphere); however, increasing towards mid-log-phase, concurring with the time-point where CO_2_ and H_2_ accumulation is maximal (Fig. 3). These genes belong to the methyl-branch of the WL, and rPFOR/Pfl C1-assimilation pathways. Among the four annotated hydrogenase genes, two displayed the highest expression levels: the correlation between expression and hydrogen oscillation/evolution suggested that Cbei_3013 and Cbei_1773 are primarily used for H_2_ uptake and H_2_ evolution, respectively. Additionally, the genes encoding for putative carbonic anhydrases also showed expression (Cbei_4425/Cbei_1031). This enzyme allows for even more intracellular CO_2_ availability^28^. This *in-silico* analysis provided an indirect overview of the gas oscillations at the transcriptomic level, while suggesting that these gases may regulate the C1-assimilatory phenotype in *C*. *beijerinckii*. Similar gas-dependent behavior has been previously observed in cultures of acetogens *Clostridium thermoautotrophicum* (reclassified as *Moorella thermoautotrophica*)^29^ and *C*. *ljungdahlii*, which contain a complete WL pathway^30,31^.

### Functional evaluation shows inorganic carbon capture by *C*. *beijerinckii*

Interested in direct evidence of C-1 assimilation, we performed mixotrophic (sucrose 3% and fructose 1.5% [w/v]) chemostat fermentations (D = 0.135 h^−1^) while steadily sparging CO_2_ and H_2_ at high and low concentrations, balanced with nitrogen. We observed steady-state consumption of CO_2_ and H_2_ along with proportional increases of apparent product-yield values above theoretical levels (Fig. S4 and Table S2). If sucrose and fructose were the only carbon and energy sources, apparent yields should have remained at or below the theoretical maximum (i.e. 0.66% C-mol, as a result of one decarboxylation from C-3 pyruvate to the C-2 acetyl group of acetyl-CoA). The higher-than-maximum apparent yields indicated additional carbon assimilation that was only possible by inorganic carbon capture.

**Figure 4:**
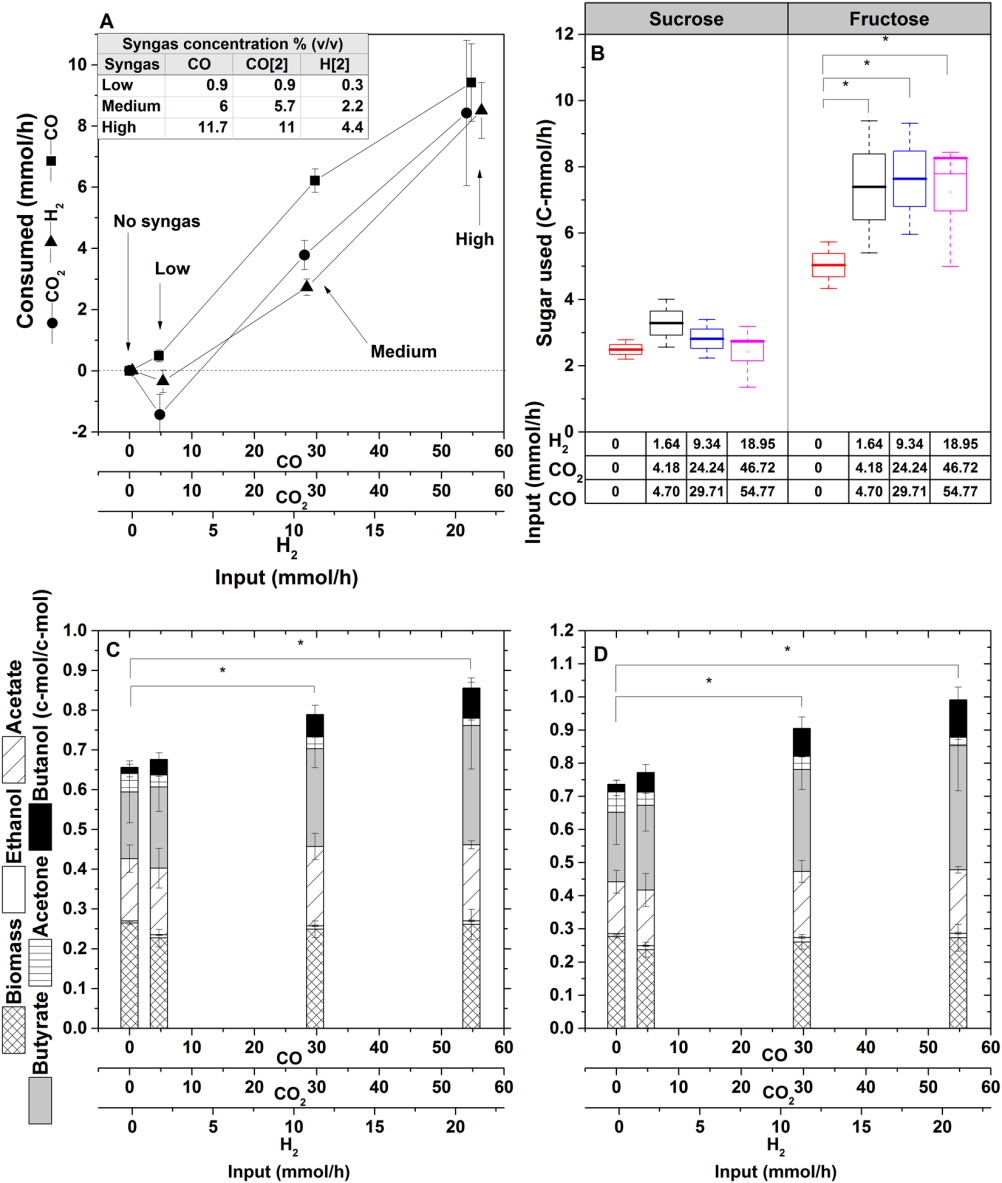
Kinetic, yield parameters and carbon and energy balances of steady-state cells (D = 0.135 h^−1^) cultured mixotrophically on presence of synthesis gas (CO, CO_2_ and H_2_). (**A**) Each tested gas mixture was constantly sparged at 12.48 L/h. Steady-state values of gas generation (H_2_ and CO_2_) and synthesis gas utilization were obtained after subtracting the net values of H_2_ and CO_2_ generated under sparged nitrogen from the values of output gas phase for each condition. Zero value indicates input = output. Negative values indicate more gases being produced than input. Positive values indicate the steady-state amounts continuously assimilated. (**B**) Sucrose and fructose consumed in steady states under the different tested gas phase condition. (**C**) Apparent yield (C-mol product/C-mol carbon source utilized) and (**D**) carbon and energy balance, calculated as $\sum (\frac{Yp.\gamma p}{\gamma s})+\,\frac{Yx.\gamma x}{\gamma s}=1$, where Yp and Yx represent C-mol ratios on figure (**C**); γ represents electrons available for each fermentation product^58^. Synthesis gas mixture was balanced with nitrogen (100% synthesis gas contains 20% CO, 20% CO_2_, 10% H_2_ and 50% N_2_). The results presented here were obtained from three biological replicates and the represented means are values at steady-state from at least three samples extracted at different retention time intervals. Significance at 0.05 refers to comparisons between whole columns.

**Figure 5:**
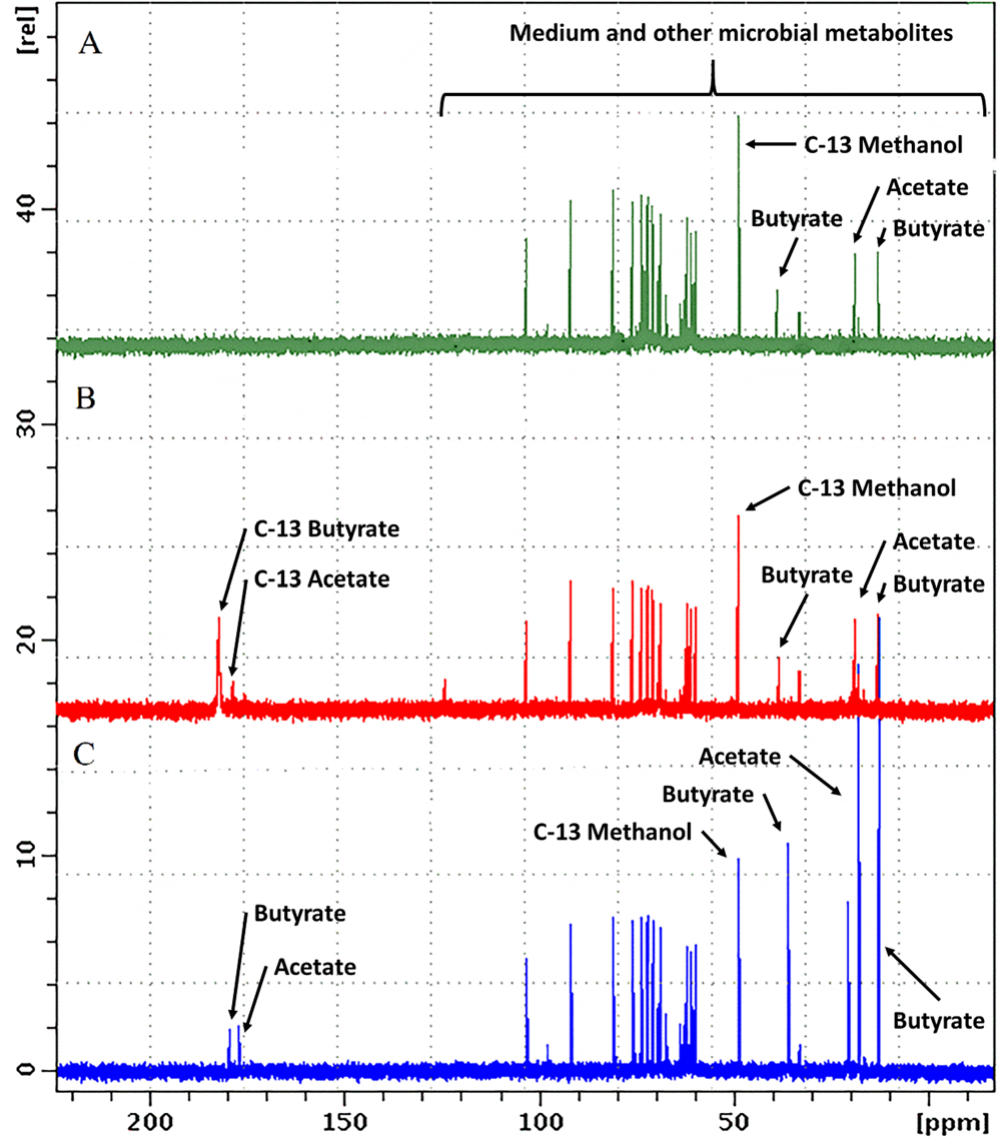
NMR spectra of C-13 labeled products. Cultures of *C. beijerinckii* SA-1 growing mixotrophically and exposed to high synthesis gas concentration (60% synthesis gas 40% N_2_, where 100% synthesis gas contains 20% CO, 20% CO_2_, 10% H_2_ and 50% N_2_) were collected at steady state (D = 0.135 h^−1^) and grown in batch during 48 h in presence or absence of ^13^C labeled CO_2_. NMR ^13^C spectra of culture media from: (**A**) Control, *C. beijerinckii* grown in the presence of 100% [v/v] CO_2_ in the headspace; (**B**) *C. beijerinckii* grown in the presence of 100% [v/v] ^13^C labeled CO_2_ tracer in the headspace; (**C**) Culture media from control cultures supplemented with standards butyric (9 µg) and acetic acid (7.68 µg). All samples contained as internal standard 0.081 µg of C-13 methanol.

Considering current efforts to transform surplus synthesis gas into biofuels^6,13^, we also sparged this gas at increasing step-wise concentrations (Table S3). Specifically, we sparged synthesis gas mixtures from low (9%), to medium (32%), to high (60%) concentrations, balanced with nitrogen (100% synthesis gas contained 20% CO, 20% CO_2_, 10% H_2_ and 50% N_2_). At low concentration, *C*. *beijerinckii* oxidized CO, releasing H_2_ and CO_2_ as shown by the steady-state values (Fig. 4A, Table S4). Accordingly, there was no significant difference in apparent C-mol yields and carbon-energy recovery balance compared to the control. Additionally, with increasing electron sink availability, the steady-state sugar utilization improved, resulting in higher product titers (Fig. 4B). Similar behavior has been observed by acetogens *Clostridium thermoaceticum* (Now *Moorella thermoacetica*)^32^, *C*. *autoethanogenum*, *Rhodopseudomonas gelatinosa* and also *Carboxydothermus hydrogenoformans*, according to the following reaction: CO + H_2_O → CO_2_ + H_2_, which is mainly used for redox balance when grown mixotrophically^12,22,33^. Therefore, in agreement with our transcriptomic analysis (i.e. overexpression of C-1 capture genes when CO_2_/H_2_ were maximal), this physiological behavior suggests these gases may be growth-limiting factors during C-1 assimilation by *C*. *beijerinckii*. Accordingly, when cultures were exposed to higher synthesis gas concentrations, higher-than-theoretical apparent C-mol yields and carbon/energy mass balances were detected with the concomitant increased consumption of H_2_, CO, and CO_2_, (Fig. 4A,C,D). Specifically, at medium and high synthesis gas concentrations, 11 and 17% more carbon, and 19 and 27% more carbon and electrons, respectively, were recovered. Interestingly, butanol and butyric acid increased by 5.5- and 1.85-fold, respectively, while biomass did not change significantly, which is typical for C1-assimilation pathways such as WL, or rPFOR/Pfl. To confirm gas assimilation into products, we collected steady-state cells (D = 0.135 h^−1^) growing under high synthesis gas concentration and cultured them in batch conditions in the presence of pure ^13^CO_2_ in the headspace as tracer. After 48 h of incubation and NMR analysis, peaks at ~ 178 and 180 ppm revealed the presence of ^13^C-labeled acetate and butyrate, respectively (Fig. 5). These peaks were likely from the enriched ^13^C quaternary carbon of these compounds. We confirmed this by adding acetate and butyrate standards (12.8 and 15 g/L, respectively), and observed an increase of peaks at 178 and 180 ppm (quaternary Cs of acetate and butyrate), at 22 (primary C of acetate), and 14 and 40 ppm (primary, secondary (also at 14 ppm), and tertiary Cs of butyrate, respectively). This is a typical spectrum of unlabeled compounds, where quaternary Cs are difficult to detect, as seen in the control in panel A (Fig. 5A). Overall, this provides direct evidence of C-1 assimilation by *C*. *beijerinckii*.

Although butanol is the main target in ABE fermentation and the proportion of total carbon in the form of *n*-butanol increased by 92%, butyric acid is also a value-added product and can be re-assimilated into *n*-butanol through multi-stage fermentations^20,34^. The generation of C-4 compounds, such as butyric acid and butanol, require more NADH than C-2 compounds (such as ethanol)^16^, underscoring the cells emphasis in recycling electrons.

The rate of gas assimilation, larger than the saturation values in each condition, also indicated biological activity (Figs 4A and S5). We also tested higher synthesis gas concentrations (up to 100%, containing 20% CO, 20% CO_2_, 10% H_2_ and 50% N_2_), showing carbon capture but generating lower yields, possibly due to carbon monoxide poisoning^35^ (Fig. S6). This indicates that the fermentation working-window for mixotrophic capture of synthesis gas by *C*. *beijerinckii* is between 30 and 60% (balanced with N_2_).

We have also performed batch fermentations of *C*. *beijerinckii* under a continuous flow of high synthesis gas concentration as sole carbon and energy source. We observed transient cell proliferation and CO assimilation (not shown). However, cell growth and gas assimilation stopped in early exponential growth phase, as the cells initiated sporulation. As a result, no products were detected.

### Transcriptomic analysis of the partial WL and rPFOR/Pfl pathways in *C*. *beijerinckii*

**Figure 6:**
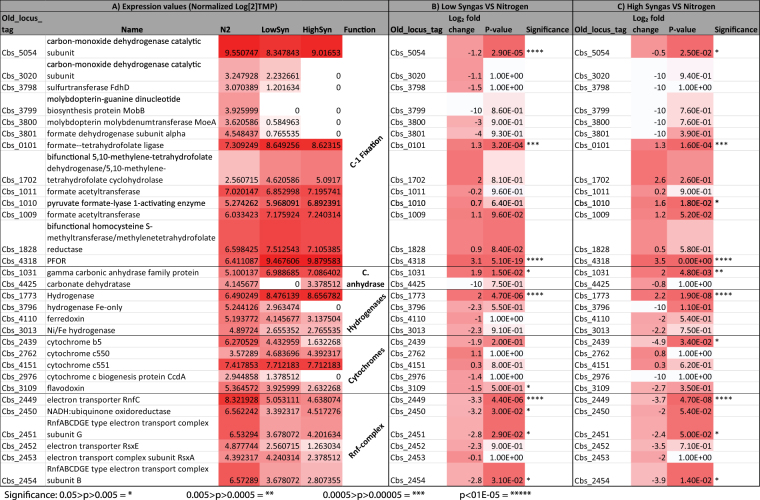
Transcription analysis of *C. beijerinckii* C-1 assimilation and energy conservation pathways. RNA was extracted from cultures growing in chemostat (D = 0.135 h^−1^) sparged (12.48 L/h) either with N_2_, low (9%), or high (60%) concentrations of synthesis gas. Cells were cultivated, in defined media^20^, 250 rpm, 37 °C and pH 6.5. (100% synthesis gas contains 20% CO, 20% CO_2_, 10% H_2_ and 50% N_2_).

To complement the time-course transcriptomic data previously described, we performed a RNA-seq experiment using chemostat cultures (D = 0.135 h^−1^) of *C*. *beijerinckii* SA-1, continuously sparged either with nitrogen (control), low or high synthesis gas. Figure 6 shows constitutive expression of each gene under N_2_ conditions (normalized to transcripts per million [TPM]), but differentially expressed under both synthesis gas conditions. Under low concentration of synthesis gas, there was a significant overexpression of a putative formate-THF ligase (Cbs_0101), which belongs to the WL pathway, a PFOR (Cbs_4318), a carbonic anhydrase (Cbs_4425), and a hydrogenase (Cbs_1773). Conversely, there was a repression of a putative CODH (Cbs_5054), a gene that belongs to the formate dehydrogenase complex (Cbs_3799), a flavodoxin (Cbs_3109), and several genes that putatively code for the Rnf-complex (Cbs_2449/54). Under high concentration of synthesis gas, there was a significant overexpression of the same genes under low synthesis gas, and a Pfl-activating enzyme gene (Cbs_1010), which belongs to the rPFOR/Pf pathway. However, under that condition, the formate dehydrogenase complex, a CODH (Cbs_3020), a hydrogenase (Cbs_3796), and a cytochrome *c* biogenesis coding protein (Cbs_2976) were completely shut down. Overall, this transcriptomic data suggests that *C*. *beijerinckii* constitutively expresses its putative genes associated to C-1 capture, by preferentially activating those belonging to the rPFOR/Pfl pathway. Specifically, when cultured under synthesis gas, the repression of the formate dehydrogenase, and the overexpression of PFOR and Pfl suggest that CO_2_ is assimilated via rPFOR/Pfl pathway (and the carbonic anhydrase), as observed with *C*. *thermocellum* ^2^.

### Nitrite as an electron sink for energy conservation

**Figure 7:**
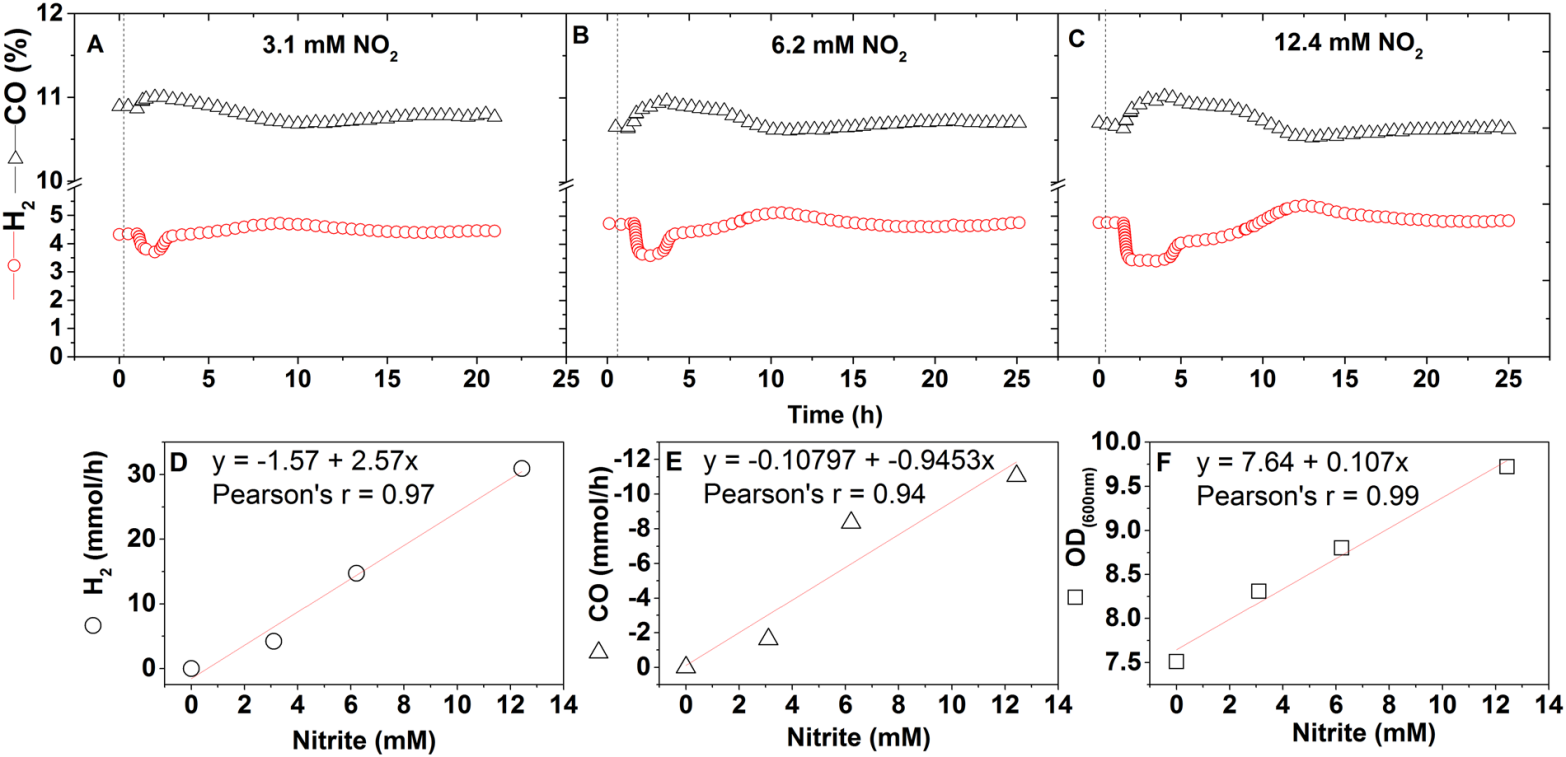
Transient responses to nitrite pulses by *C. beijerinckii* growing in chemostat (D = 0.135 h^−1^). Experiments were performed on defined medium^20^ containing 3% sucrose and 1.5% fructose (w/v) and sparged with 60% (v/v) synthesis gas balanced with nitrogen (100% synthesis gas contains 20% CO, 20% CO_2_, 10% H_2_ and 50% N_2_) at 37 °C. The additions of sodium nitrite are indicated with vertical dashed lines to reach final concentrations as follow; 3.1 mM (**A**), 6.2 mM (**B**) and 12.4 mM (**C**). The CO and H_2_ data shown were obtained by monitoring, in real time, with an EasyLine continuous gas analyzer, model EL3020 (ABB, Germany). Nitrite concentrations higher than 24 mM proved toxic and led to washout. Steady-state values were re-established prior to testing each nitrite concentration. Correlations of NO_2_ added with (**D**) H_2_ consumed, (**E**) amount of CO consumption displaced, and (**F**) biomass increase, were calculated from the slopes after fitting the data to linear regressions.

Under mixotrophic growth, the C-1 assimilation pathways operate mainly for electron recycling^2,22,36^. Consequently, an alternative way to demonstrate an active pathway is to inhibit CO assimilation by providing an alternative and preferred electron acceptor. Both nitrate and nitrite are known to have this effect on CO assimilation by acetogenic bacteria^37,38^. To test this hypothesis in *C*. *beijerinckii*, we performed chemostat pulse experiments under high synthesis gas concentration (i.e. 60% [v/v]). Interestingly, nitrate showed no effect on *C*. *beijerinckii*. However, less-reduced nitrite partially inhibited CO assimilation (1 mol per mol of NO_2_), while increasing hydrogen consumption (2.5 mol of H_2_ per mol of NO_2_). Additionally, biomass increased proportionately, shifting the pathway from catabolism to anabolism (Fig. 7). Both electron acceptors have also been shown to increase biomass in acetogens *C*. *thermoautotrophicum* and *Moorella thermoacetica* ^37,38^. The H_2_-dependent CO_2_, or CO assimilations are thermodynamically unfavorable as they do not generate a gain in ATP^36^. Thus, nitrite reduction is preferred as a less expensive way to recycle electrons. As such, the nitrite reductase reaction requires only electrons, in the form of hydrogen and reduced ferredoxin (i.e. not ATP). *C*. *beijerinckii* contains a putative ferredoxin-nitrite reductase (Cbei_0832), likely responsible for the observed phenotype, which also unveils this species as a facultative nitrite dissimilator.

### Transcription of alternative energy-conservation genes

Considering that there is no net ATP generation through C-1 assimilation pathways, autotrophic bacteria rely either on substrate-level phosphorylation or on chemiosmosis for energy conservation^7,22,36^. Examples of the latter include cytochromes, Na^+^ pumps, or the Rnf-complex, whereby acetogens generate an ion gradient for energy generation through ATP-synthases. It has been suggested that *B-*type cytochromes are responsible for H^+^-dependent ATP generation, and can be coupled to a membrane-bound methylene-THF reductase. Its location suggests the role of this enzyme in energy conservation^39^. *C*. *ljungdahlii* contains a Rnf-complex but not cytochromes^5,40,41^. Interestingly, the *C*. *beijerinckii* genome encodes cytochromes (also involved in nitrite reduction^42^) *b*-type (Cbei_2439), *c*550 (Cbei_2762), *c*551 (Cbei_4151), *c* biogenesis protein (Cbei_2976), cytochrome-bound flavoproteins (Cbei_3109), and also genes coding for the Rnf-complex (Cbei_2449-2454). Additionally, the methylene-THF reductase of *C*. *beijerinckii* is predicted^43^ to contain transmembrane domains. The transcriptomic analysis of the publicly available RNA-seq data^27^ showed high expression of all these energy-conserving genes, especially the Rnf-complex (Fig. 3). In line with this observation, our transcriptomic analysis of chemostat cultures of *C*. *beijerinckii* shows constitutive expression of these genes under nitrogen exposure, and a modest repression under low and high concentrations of synthesis gas (Fig. 6). Since sporulation prevents *C*. *beijerinckii* to growth autotrophically, these chemiosmotic mechanisms are potentially useful only during mixotrophic growth. Similarly, C. *ljungdahlii* requires the Rnf-complex when cultured mixotrophically^41^.

## Discussion

The variability on apparent product yields reported in the literature and the empirical records of microbial solvent production, demonstrated the need for a deeper study of the evolving gas-phase as signals for overlooked pathways. We have shown that *C*. *beijerinckii* captures inorganic carbon and hydrogen under mixotrophic conditions, increasing apparent product yields above theoretical heterotrophic values. Among the putative WL pathway genes, *C*. *beijerinckii* does not contain annotated an acetyl-CoA synthase, but its CODHs have Fe-S and Ni-Fe-S metal centers, which are typical of CODH subunits of bifunctional CODH/acetyl-CoA synthases^44–46^. However, it is likely that this enzyme in *C*. *beijerinckii* does not lead to acetyl-CoA synthesis, and thus autotrophic growth. Indeed, as has been recently shown, a mutant strain of *C*. *ljungdahlii* with a SNP (single nucleotide polymorphism) in its CODH gene located in its WL cluster (i.e. the one with lower sequence identity to that of *C*. *beijerinckii*, and associated to a acetyl-CoA synthase) loses its autotrophic phenotype^47^, even when its CODH with similarity to *C*. *beijerinckii* was intact. A similar phenotype has been observed with a CODH-mutant strain of *C*. *autoethanogenum* ^48^. Nevertheless, *C*. *beijerinckii* contains the genetic potential for an active rPFOR/Pfl-based C-1 capture, including an additional formate dehydrogenase, not present in *C*. *thermocellum* ^2^.

**Figure 8:**
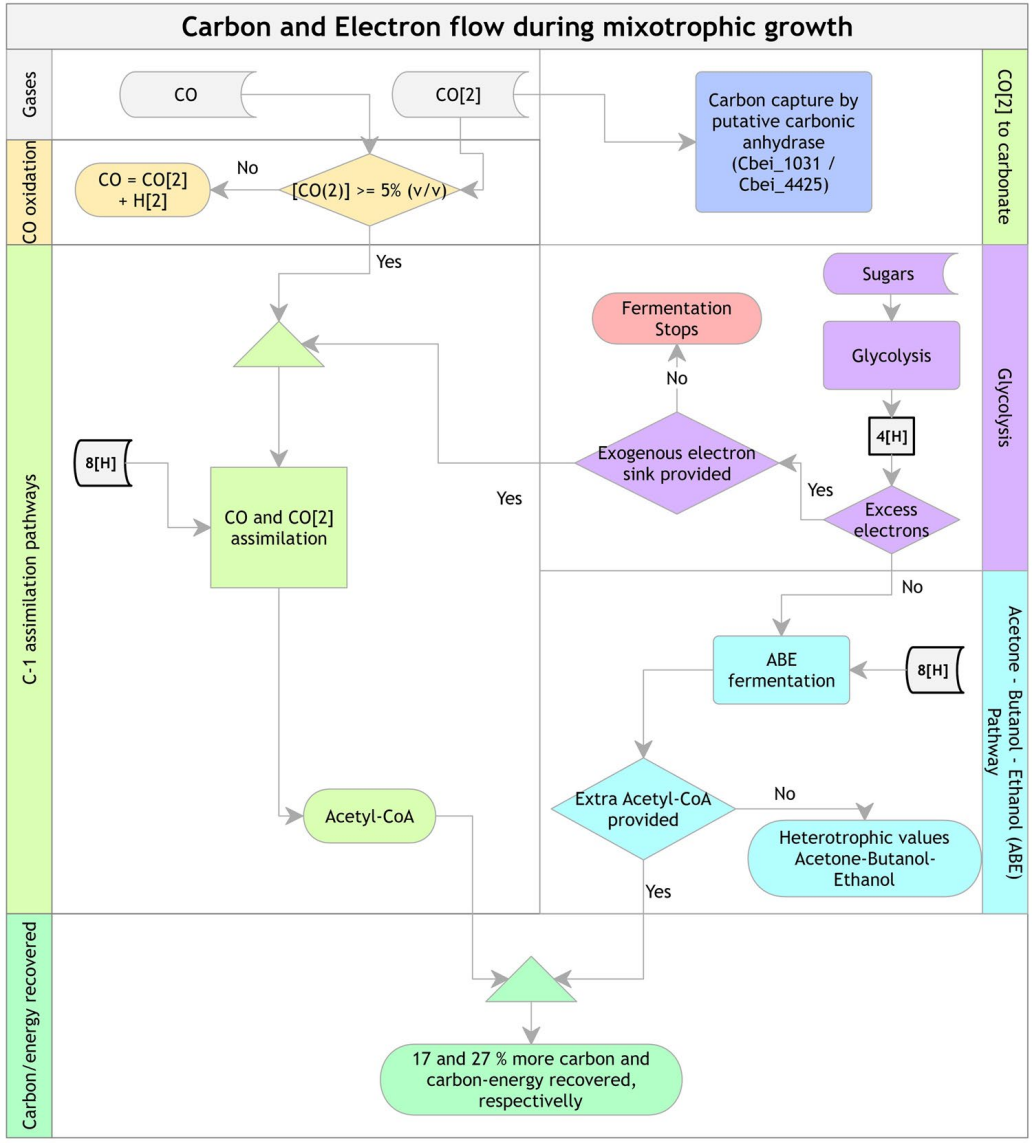
Logic model of carbon-electron flow in *C*. *beijerinckii* grown mixotrophically. The data suggest that in the presence of CO and CO_2_, there are three possible paths for carbon capture: 1) CO_2_ to carbonate through putative carbonic anhydrase, or 2) CO oxidation to generate CO_2_ + H, if the CO_2_ in the gas-phase is < 5% (v/v); or finally, 3) the C-1 assimilation, if CO_2_ > 5%. Simultaneously, supplied sugars proceed to glycolysis. In the absence of an electron bottleneck, ABE-fermentation utilizes all the sugar-derived carbon and electrons. Otherwise, and if no external electron sink is provided, fermentation stops. In the presence of an external electron sink (such as CO/CO_2_), #2 or #3 take place. If #3 takes place, the extra acetyl-CoA generated, along with the still-running ABE-pathway, leads to up to 17 and 27% more carbon and carbon-energy recovered, respectively.

Based on our physiologic data, we propose a logic model to explain the carbon-electron flow during mixotrophic growth of *C*. *beijerinckii* cultures (Fig. 8). In the presence of CO and CO_2_, there are three possible paths for carbon capture: 1) CO_2_ to carbonate through putative carbonic anhydrase, or 2) CO oxidation to generate CO_2_ + H_2_, if the CO_2_ in the gas-phase is <5% (v/v); or finally, 3) C-1 assimilation into acetyl-CoA, if CO_2_ > 5%. Simultaneously, supplied sugars proceed to glycolysis. In the absence of an electron bottleneck, ABE-fermentation utilizes all the sugar-derived carbon and electrons. Otherwise, and if no external electron sink is provided, fermentation stops, limiting sugar utilization. In the presence of an external electron sink (such as CO/CO_2_), #2 or #3 takes place. If #3 takes place, the extra acetyl-CoA generated, along with the still-running ABE-pathway, leads to 17 and 27% more carbon and carbon-energy recovered, respectively. Moreover, this physiological capability improves product titers by increasing sugar utilization. However, it is important to note that under our experimental conditions, we recovered an apparent ~86 and 100% total carbon and carbon/electron, respectively.

Mixotrophic C-1 assimilation was previously shown by cultures of acetogen *C*. *ljungdahlii*, whereby exogenous CO_2_ gas increases carbon recovery^49^. The discovery of the same phenotype by cultures of *C*. *beijerinckii* has important implications in our understanding of the biology of this industrial butanol-producer, and adds a new alternative for greenhouse gas-capture. Indeed, *C*. *beijerinckii* stands out among traditional solventogenic species because: (i) it contains genetic elements for cytochromes and the Rnf-system; (ii) it contains genes that code for catalytic enzymes that belong to the WL (except acetyl-CoA synthase) and rPFOR/Pfl pathways; and (iii) the synchronous H_2_/CO_2_ oscillation is an example of a natural integrated oscillator, that can potentially be used for feedback controls in biosensors^50,51^. Future work involving knockout/complementation and biochemical studies will expand our understanding of these processes. Our approach for in-line endogenous gas monitoring shows that it can readably be utilized to uncover new pathways, or potentially even survey a culture (or consortia) for volatile metabolic signatures, in real-time.

## Materials and Methods

### Organisms

*Clostridium beijerinckii* SA-1 (ATCC 35702)^26^ was obtained from the American Type Culture Collection (ATCC). Its identity was verified by PCR amplification and sequencing of the 16S rRNA gene using the prokaryotic 16S rDNA universal primers 515F (5-GCGGATCCTCTAGACTGCAGTGCCA-3) and 1492R (5-GGTTACCTTGTTACGACTT-3).

### Bacterial medium and inoculum preparation

*C*. *beijerinckii* stocks were activated as previously described^17^ and were grown in a previously designed medium^20^. The base components were autoclaved and the sugar (6% w/v sucrose) and trace components were added aseptically to the medium reservoir by filtration (0.22 µm). The inocula were prepared as consistently performed by our lab^52^. Exact fermentation conditions are detailed in the Main Text section.

### Bacterial culture conditions

Growth experiments were performed in fed-batch or chemostat modes of operation in a 2-Liter Biostat^®^ B plus fermenter equipped with controllers for pH, temperature, agitation, and gas mass-flow (Sartorius BBI Systems, Germany). The temperature was set at 37 °C, agitation speed at 250 rpm, and pH 6.5 by the automated addition of 0.5 N KOH or 25% (v/v) H_3_PO_4_, into a working final volume of 1,400 mL of culture for fed-batch or 700 mL for chemostat. The fed-batch fermentations were started with 6% (w/v) sucrose, and 400 mL containing 80 g of the same sugar were added at constant feed rate (0.08 mL/h) to reach a final concentration of 100 g/L (w/v). The initial volume was 1 L and final 1.4 L. Exact feed components and times of feed start are detailed in the Main Text section. For the chemostat experiments the conditions were identical as described for fed-batch except the carbon and energy source were 3% (w/v) sucrose and 1.5% fructose. Once the cells reached exponential phase under sparged pure nitrogen (OD600 nm ~ 1), the feed and harvest flow were initiated and adjusted to a dilution rate D = 0.135 h^−1^. Exact sparged gas compositions are detailed in the Main Text section, steady-state conditions were verified for each condition and at least three retention times were allowed before sampling was initiated. Three samples at each steady-state condition were obtained from at least one retention time intervals. The discrete ratios of continuous gas streams were always sparged at 12.48 L/h. Different gas-phase conditions, from pure nitrogen gas to increased synthesis gas concentrations, were achieved by modifying the mix ratios between synthesis gas and nitrogen using two mass flow controllers; the exact concentrations tested are detailed in the Results section (and Table Supplemental 2). Inlet and exhaust gases in the gas-phase (O_2_, N_2_, CO, CO_2_, H_2_, and Ar) were monitored and recorded in real-time using in-line O_2_/CO_2_ and H_2_/CO EasyLine continuous gas analyzers, model EL3020 (ABB, Germany), and a Pfeiffer OmniStar quadrupole mass spectrometer. Biomass proliferation in the fermentation tank was monitored and recorded using an in-line biomass sensor (Fundalux, Sartorius, BBI Systems, and Germany) and also by (SmartSpec Plus, BioRad, USA). Dry weight concentration was obtained by filtering a portion of sample using vacuum suction through a 0.2-µm-pore-size filter of known mass (mixed cellulose esters; EMD Millipore, Germany); the filter was then dried at 60 to 70 °C for 7 days and reweighed until constant weight. Pre-mixed synthesis gas was obtained from National Welders in Goldsboro, NC, USA.

### Sample analysis

Sucrose, fructose, acetic acid and butyric acid were quantified with a high-performance liquid chromatograph (HPLC) under isocratic conditions at 65 °C, and a mobile phase of water at a 0.5 mL/min flow rate using a Supelcogel TM Ca column (300 mm × 7.8 mm, Supelco TM Analytical, Bellefonte, PA, USA) coupled to a refractive-index detector. Solvents (acetone, butanol and ethanol) were separated in a gas-chromatograph (GC) SS Porapak Q 80/100 column (OV, Marrietta, OH, USA) in a GC (GC-8A) fitted with a flame ionization detector (FID) (Shimadzu Corporation, Kyoto, Japan), using 200 kPa of nitrogen as the mobile phase with an injection temperature of 220 °C and a column temperature of 140 °C.

C-13 labeled-CO_2_ experiments were performed using 50 mL of culture was collected from the steady state (D = 0.135 h^−1^) under high synthesis gas concentration (11.17% CO, 11.01% CO_2_ and 4.40% H_2_ [v/v]), depicted in Fig. 4. Cultures were incubated for 48 h in sealed 250 mL serum bottles containing 100% C-13-labeled CO_2_ in the head space, at 37 °C, 200 rpm. For analysis, samples were sent to the NMR-Center of the Chemistry Department at NC State University. 13-C NMR spectra were carried out using a Bruker DRX-500 spectrometer equipped with a 5 mm ITD probe, maintaining the temperature constant at 298 K and the acquired process data were measured with the same parameter. The “zgig” pulse sequence was used. The data were acquired with 2600 FIDs. The internal standards used were C-13 methanol, butyric and acetic acid (0.081, 9.051 and 7.68 µg, respectively), into 600 µL of sample, diluted into 10% D2O).

### Proteins sequence identity analysis

Protein sequence identities were performed as previously described^53^.

### RNA-seq analysis from Wang *et al*

The sequence reads from the transcriptional profiling experiment of Wang *et al*.^27^ were downloaded from the NCBI Sequence Read Archive (SRA045799) and imported into the Cyverse Collaborative Discovery Environment^54^. The sequence reads were quality filtered with the trimmomatic program^55^ using the trimmers, “LEADING:5 TRAILING:5 SLIDINGWINDOW:4:15 MINLEN:36”. Two independent platforms were subsequently used to analyze these normalized data, Cyverse Discovery Environment and Geneious v9 (Biomatters Ltd., New Zealand), while aligning the sequences to the *C*. *beijerinckii* NCIMB8052 genome (GenBank accession CP000721.1). In Cyverse, the sequences were aligned with tophat2^56^ using the default parameters, while differences in transcript abundance were determined using the Cuffdiff program, which is part of the Cufflinks software package^57^. The analyses in Geneious were performed using default parameters.

### Transcription expression analysis

To complement the time-course transcript expression analysis of RNA-seq experiments previously described, we performed chemostat cultures (D = 0.135 h^−1^) of *C*. *beijerinckii* SA-1 growing in presence of sparged N2, low, or high synthesis gas (Fig. S4). For each condition cultures growing in steady-state were harvested (10 mL) and immediately flash frozen and sent for RNA extraction and sequencing at the Microbiome Core Facility at the University of North Carolina Chapel Hill, NC.

### RNA isolation

RNA from bioreactor-derived bacteria was isolated using PowerMicroBiome RNA isolation kit from MO Bio Laboratories (San Diego, CA). Briefly, the bacteria pellets were combined with lysis buffer and glass beads. Subsequently they were lysed for 5 minutes in Qiagen TissueLyser II (Valencia, CA) at 30 Hz. Further, the process included inhibitor removal step and standard on-column purification was carried out according to manufacturer’s instructions. RNA purification included on-column DNAse treatment for 15 minutes at room temperature. Subsequently RNA concentration and quality were determined by RNA electrophoresis on Agilent bioanalyzer (Santa Clara, CA).

### rRNA removal and library preparation

rRNA was removed using Illumina Ribo-Zero Gold Bacteria Kit (San Diego, CA), according to manufacturer’s instructions. Briefly, the rRNA-specific magnetic beads were washed with storage buffer and were mixed with 500 ng of total sample RNA. Subsequently, rRNA removal solution was added and samples were incubated for 10 minutes at 65 °C. Finally, samples were placed on a magnetic stand for 15 minutes in room temperature and coding RNA was aspirated after which it was immediately preceded to mRNA library preparation protocol. We used Illumina TruSeq Stranded mRNA Library Prep Kit (San Diego, CA), according to manufacturer’s instructions. Briefly, RNA was mixed with Fragment-Prime mix and incubated at 94 °C for 8 minutes and then it was immediately subject to first strand and second strand cDNA synthesis reactions, respectively, followed by 3′ end repair, adenylation and adapter ligation. After adapter ligation, the libraries were enriched by polymerase chain reaction using the following thermal cycling conditions: 98 °C for 30 s, followed by 15 cycles of 98 °C for 10 s, 60 °C for 30 s and 72 °C for 30 s. Final extension step of 70 °C for 5 minutes was also carried out. After enrichment by PCR, libraries were purified with Beckman Coulter magnetic beads (Brea, CA) followed by an 80% ethanol wash, validated on Agilent bioanalyzer and DNA concentration was determined using Quant-iT PicoGreen dsDNA Reagent from Thermo Fisher Scientific (Eugene, OR).

### RNA-seq analysis

Reads from two separate sequencing runs were concatenated to maximize sequencing depth and coverage. RNA-Seq data were analyzed using CLC Genomics Workbench v9.5 (QIAGEN Bioinformatics, Redwood City, CA). Paired-end reads were combined and reads were trimmed of any remaining adapter sequences using CLC’s Illumina read import feature using default parameters. The *Clostridum beijerinckii* SA-1 genome (GenBank accession number CP006777) was downloaded from NCBI using the CLC GenBank browser. The SA1 nucleotide sequence was then converted into a genome track and the associated annotations were used to create a track for gene evidence. All reads were then mapped to the reference genome using the CLC RNA-Seq analysis feature with default parameters. Expression level data were reported as transcripts per million (TPM). Finally, differential expression analyses were performed using CLC’s Advanced RNA-Seq plugin. The data generated in these analyses allow for the generation of volcano plots in OriginPro 2015 graphing software (OriginLab Corporation, Northhampton, MA). Bam files were deposited in NCBI (PRJNA390299).

### Gas calculations

For gas solubility, we used Henry’s law: *C* = *k *× *p* where: C is concentration, k is Henry’s constant at 37 °C and p is partial pressure.

The k values used were (in g/L): 0.0225 for CO; 1 for CO_2_; 0.033 for O_2_ and 0.0014 for H_2_.

To calculate gas consumption *C* = (*O* − *I* + *E*) × (−1), where O is output, I is input, and E is the amount the cells endogenously generated under nitrogen. Positive values indicate consumption. Negative values indicate generation.

### Stoichiometry

For this calculation we used the methods previously reported^58^.

### Nitrite pulse experiments

We performed continuous culture pulse experiments with different concentrations of nitrite in the form of sodium nitrite (Sigma-Aldrich Inc., Saint Louis, MO, USA). Exact conditions are detailed in the Main Text section.

## Electronic supplementary material

Supplementary Material
