# Supplementary material for: Evidence of mixotrophic carbon-capture by n-butanol-producer Clostridium beijerinckii
Source: Sci Rep. 2017 Oct 6;7:12759. doi: 10.1038/s41598-017-12962-8 (PMC5630571; doi:10.1038/s41598-017-12962-8)
Supplement: Supplementary file 1 — Supplementary Material [file 41598_2017_12962_MOESM1_ESM.doc]

Supplementary Materials for:

Evidence of mixotrophic carbon-capture by n-butanol-producer *Clostridium beijerinckii*

W.J. Sandoval-Espinola^1,4^, M. Chinn^2^, M.R. Thon^3^, J.M. Bruno-Barcena^1*^

correspondence to: jbbarcen@ncsu.edu

**This PDF file includes:**

Figs. S1 to S6

Tables S1 to S4

Fig. S1


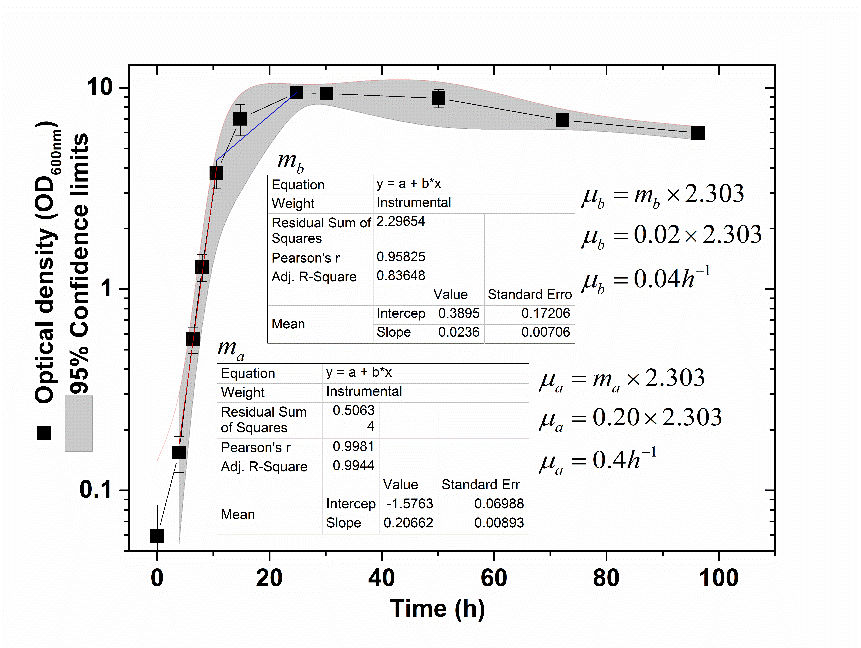


Fig. S1. Mean growth curve of multiple fed-batch fermentations of *C. beijerinckii*. Two slopes, at early exponential (m_a_) and late exponential (m_b_) growth phases, respectively, are shown. µ_a_ and µ_b_ are early and late specific growth rates, respectively.

Fig. S2


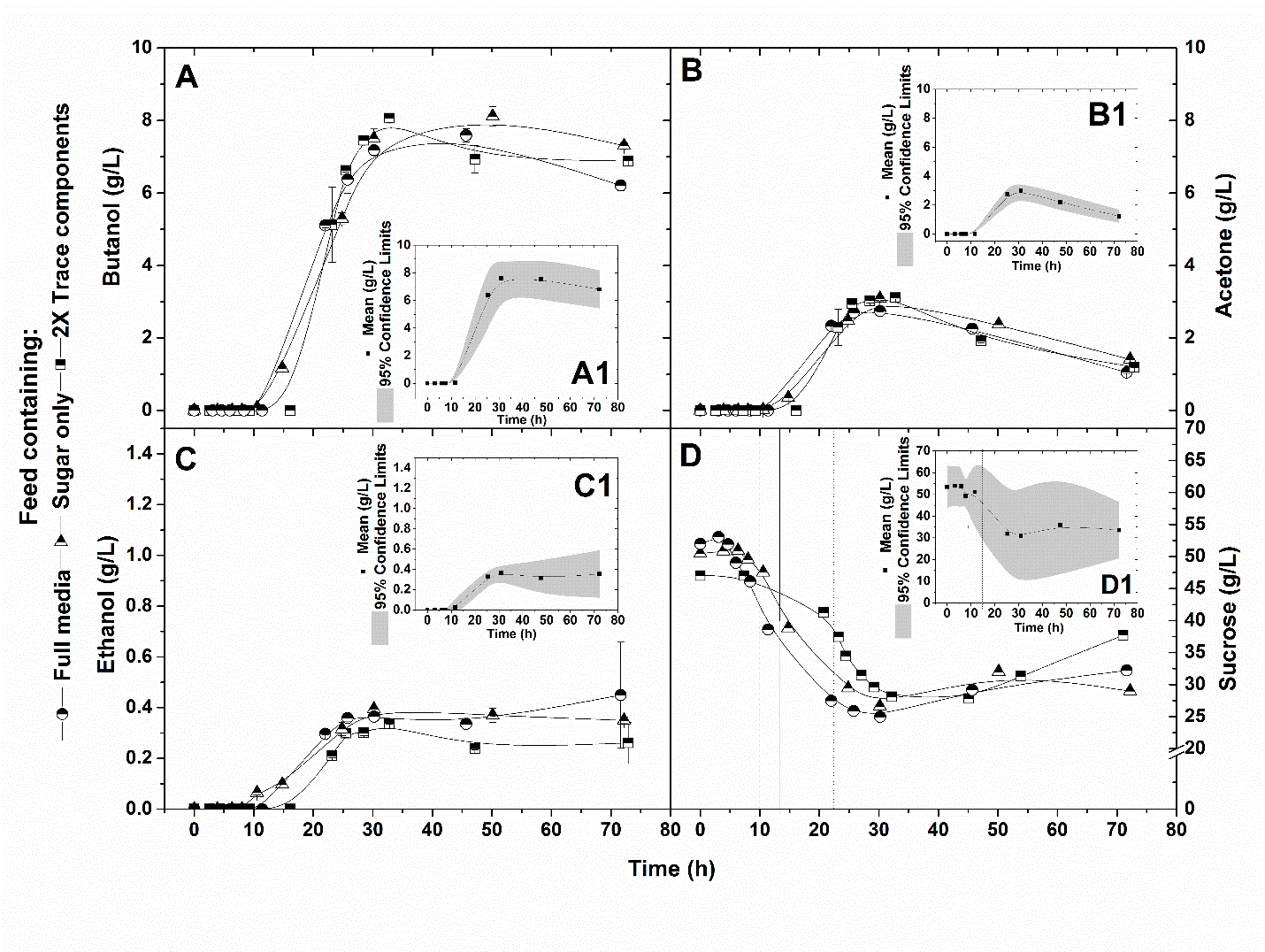


Fig. S2. Product and substrate profiles of fed-batch fermentations of *Clostridium beijerinckii*. Different feed nutritional compositions were used: Circle: whole medium + sucrose; Triangle: sucrose only and Square: 2x trace components + sucrose. Experiments were performed in a defined medium (18) containing an initial amount of 6% (w/v) sucrose as limiting carbon and energy source. The final sucrose concentration (feed + initial medium) was 100 g/L, fed at 0.08 mL/h. Temperature was 37 ˚C and pH was controlled (6.5). initial and final volumes were 1 and 1.4 L, respectively. Nitrogen gas was flowed filtered-sterilized at 12.48 l/h throughout each experiment. A: Butanol; B: Acetone; C: Ethanol and; D: residual sucrose. A1, B1, C1 and D1 show the mean values and 95% confidence limits of butanol, acetone, ethanol and sugar, respectively. The vertical lines in D represent the time at which each feed was started: solid: whole medium + sucrose; dashed: sugar only and; dots: 2x trace components + sucrose. The solid vertical line in D1 represents the mean time at which feed was started. Error bars indicate SD.

Fig. S3

**
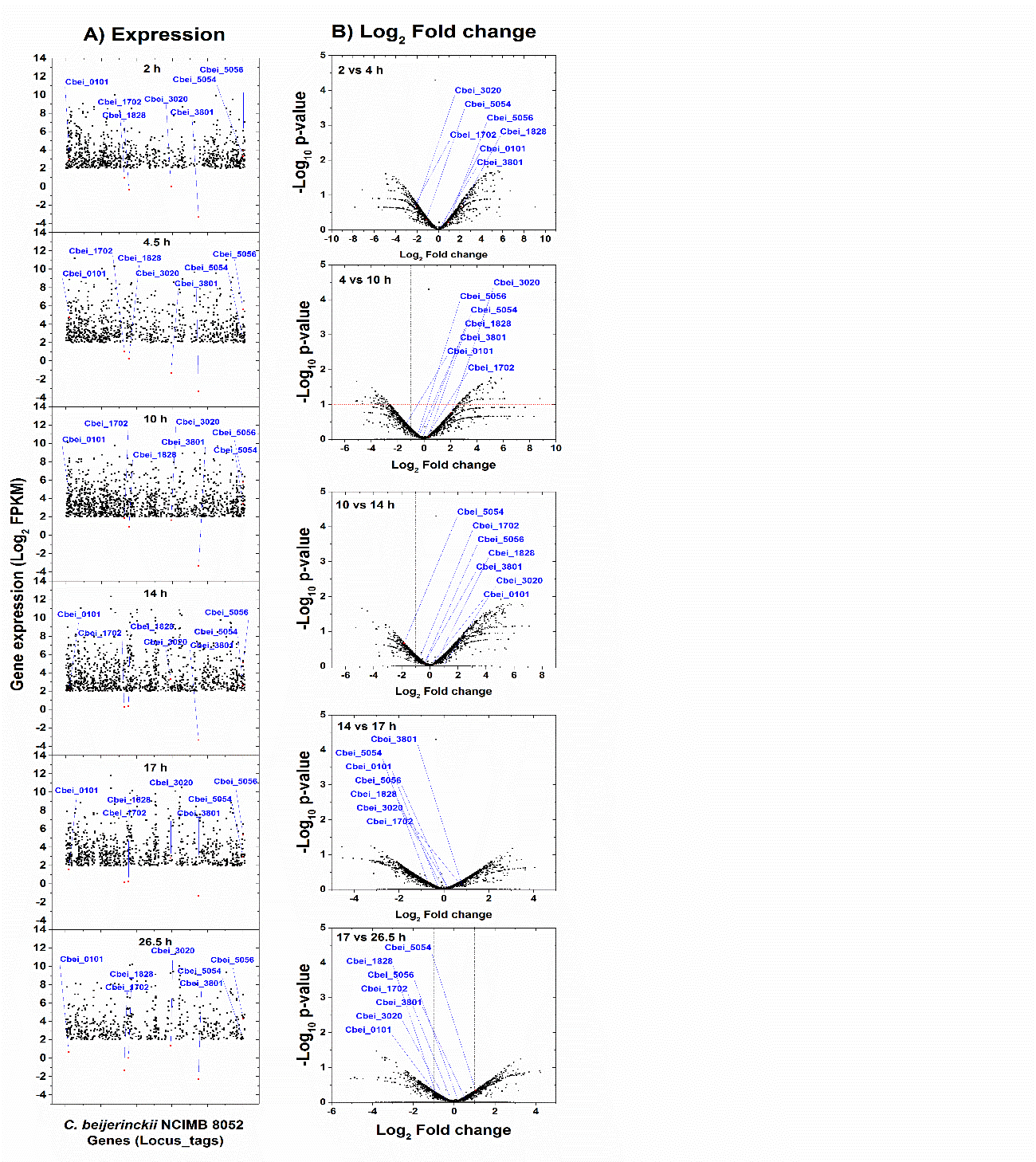
**

**Fig. S3. Time-point genome-wide expression profile and comparison of *C. beijerinckii* NCIMB 8052.** A) Time-point genome-wide expression levels (log_2_ of fragment per kilo base per million -FPKM-), minus housekeeping genes (HKG) and those whose expression were below log_2_ = 2, in *C. beijerinckii* NCIMB 8052, highlighting Wood-Ljungdahl (WL) pathway genes. B) Volcano plots comparing expression levels of each time point. Alpha: 0.1; fold change considered: 1 log_2_ fold.

Fig. S4


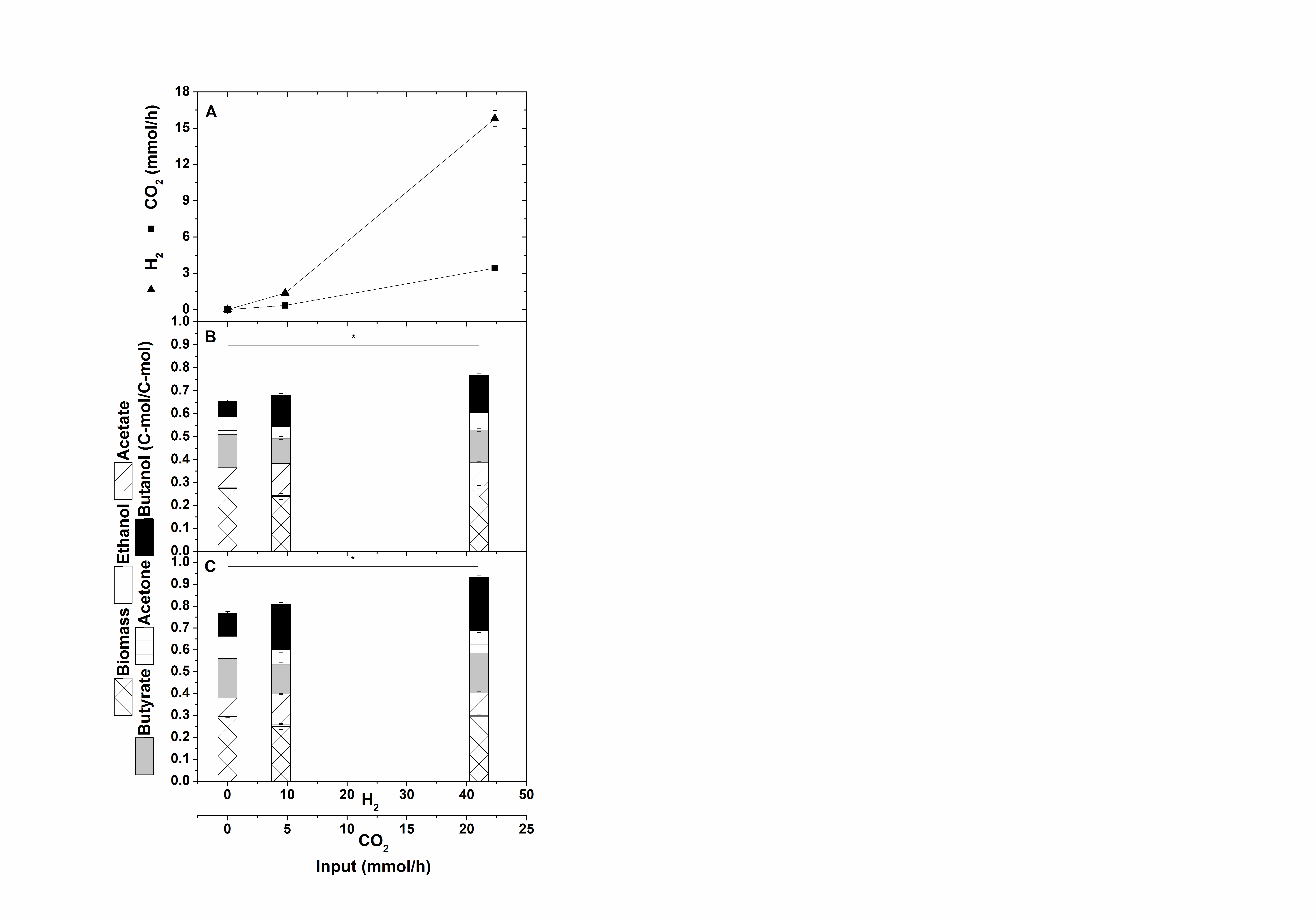


**Fig. S4. CO_2_ and H_2_ consumption, carbon recovery and carbon and energy balance.** (A) Steady-state values (D = 0.135 h^-1^) of CO_2_ and H_2_ utilization calculated as absolute values of amount of H_2_ and CO_2_ produced by the cells under nitrogen conditions plus exogenous gases minus output. Positive values indicate the amount that the cells continuously assimilate, at a flow of 12.48 L/h. (B) Yield C-mol ratio and (C) carbon and energy balance, calculated as
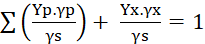
, where Yp and Yx represent C-mol ratios of each product and biomass; γ represent electrons available*(*48). The results presented here were obtained from three biological replicates and the represented means are values at steady-state conditions from at least three samples extracted at different retention time intervals. Significance at 0.05 refers to comparisons between whole columns.

Fig. S5.


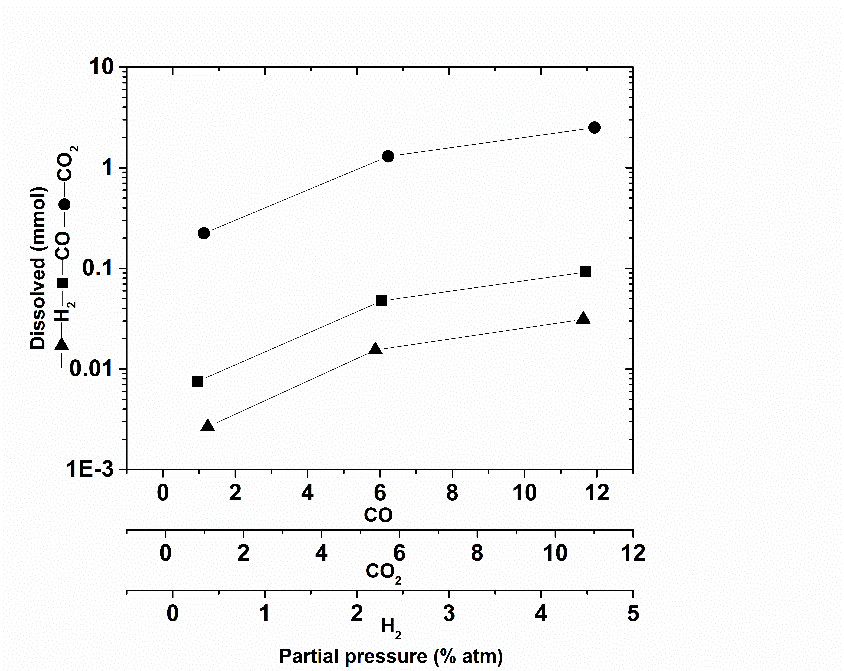


**Fig. S5. Estimation of dissolved gases in the medium.** Saturation values of each gas in the liquid phase, calculated according to Henry’s laws, for gas and liquid at 37 °C at different partial pressures.

Figure S6


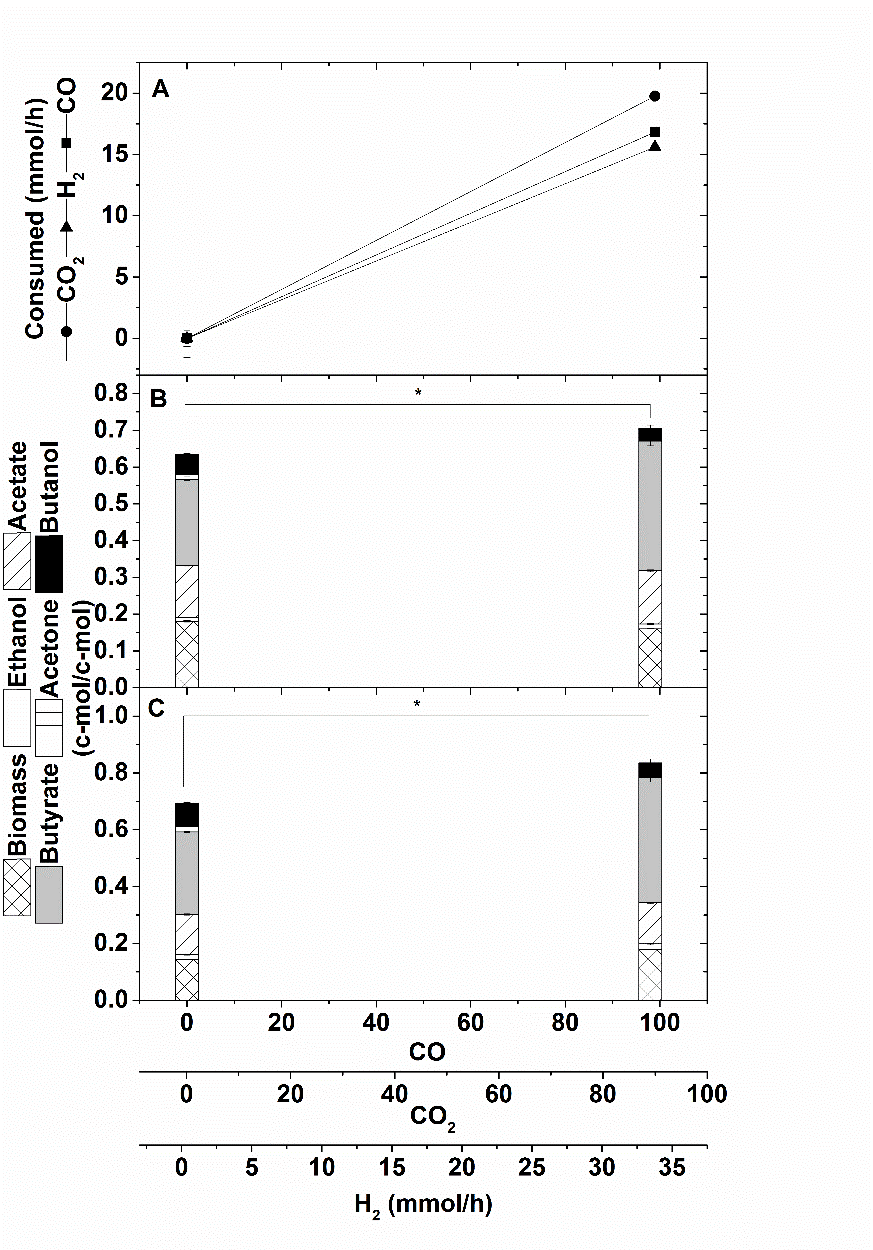


**Fig. S6. Synthesis gas (100%) consumption, carbon recovery and carbon and energy balance.** (A) Steady-state values (D = 0.135 h^-1^) of synthesis gas utilization (CO. CO_2_ and H_2_) calculated as absolute values of amount of H_2_ and CO_2_ produced by the cells under nitrogen conditions plus exogenous gases minus output. Flow = 12.48 L/h. (B) Yield C-mol ratio and (C) carbon and energy balance, calculated as
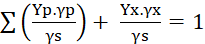
, where Yp and Yx represent C-mol ratios of each product and biomass; γ represent electrons available*(*48). The results presented here were obtained from three biological replicates and the represented means are values at steady-state conditions from at least three samples extracted at different retention time intervals. Significance at 0.05 refers to comparisons between whole columns.

Table Supplemental 1.

Table 1. Final kinetic and yield parameter. Values were obtained after different fed-batch fermentations of *Clostridium beijerinckii* at 37 ᵒC, pH 6.5 and feed at 0.08 mL/h, in defined media (18).

| **Feed containing** | **Variables and Parameters** | **Values** |
| --- | --- | --- |
|  |  |  |
| Sugar only | Biomass (OD_600_/mL) | 9.5 |
|  | Biomass yield (OD_600_/g) | 0.14 |
|  | Butanol (g/L) | 8.11 ± 0.26 |
|  | Acetone (g/L) | 2.37 ± 0.02 |
|  | Ethanol (g/L) | 0.36 ± 0.02 |
|  | Total Solvents (g/L) | 10.85 ± 0.26 |
|  | Sugar utilized (%) | 75 |
|  | Specific Growth rate µ (h^-1^) | 0.46 |
|  | Butanol yield (Y_g/g_) | 0.10 |
|  | Total Solvents yield (Y_g/g_) | 0.14 |
|  | Butanol productivity (g/L.h) | 0.16 |
| Whole Medium | Biomass (OD_600_/mL) | 10.46 |
|  | Biomass yield (OD_600_/g) | 0.17 |
|  | Butanol (g/L) | 7.59 ± 0.18 |
|  | Acetone (g/L) | 2.26 ± 0.11 |
|  | Ethanol (g/L) | 0.33 ± 0.01 |
|  | Total Solvents (g/L) | 10.28 ± 0.16 |
|  | Sugar utilized (%) | 69 |
|  | Specific Growth rate µ (h^-1^) | 0.56 |
|  | Butanol yield (Y_g/g_) | 0.10 |
|  | Total Solvents yield (Y_g/g_) | 0.14 |
|  | Butanol productivity (g/L.h) | 0.25 |
| 2x Trace components | Biomass (OD_600_/mL) | 10.7 |
|  | Biomass yield (OD_600_/g) | 0.17 |
|  | Butanol (g/L) | 8.05 ± 0.12 |
|  | Acetone (g/L) | 3.11 ± 0.03 |
|  | Ethanol (g/L) | 0.33 ± 0.002 |
|  | Total Solvents (g/L) | 11.5 ± 0.15 |
|  | Sugar utilized (%) | 66 |
|  | Specific Growth rate µ (h^-1^) | 0.48 |
|  | Butanol yield (Y_g/g_) | 0.10 |
|  | Total Solvents yield (Y_g/g_) | 0.14 |
|  | Butanol productivity (g/L.h) | 0.28 |

Table Supplemental 2.

Table 2. Carbon and carbon and energy balances of chemostat (D = 0.135 h^-1^) cultures of *Clostridium beijerinckii* SA-1 continuously sparged with CO_2_ and H_2_ (F = 12.48 L/h).

| **Gas input** | Condition | CO_2_ | H_2_ |  |  |
| --- | --- | --- | --- | --- | --- |
|  |  | mmol/h | mmol/h |  |  |
|  | Control | 0 | 0 |  |  |
|  | Low | 4.82594 | 8.94448 |  |  |
|  | High | 22.35156 | 42.04261 |  |  |
| **Biomass** | Condition (Synthesis gas) | C and e balance | | C-balance | |
|  |  | Cmol/Cmol | SD | Cmol/Cmol | SD |
|  | Control | 0.28814 | 0.00149 | 0.27507 | 0.00142 |
|  | Low | 0.24964 | 0.01405 | 0.23832 | 0.01342 |
|  | High | 0.29453 | 0.00671 | 0.28118 | 0.00641 |
| **Ethanol** | Control | 0.00739 | 4.25E-04 | 0.00493 | 2.83E-04 |
|  | Low | 0.0074 | 0.00197 | 0.00493 | 0.00131 |
|  | High | 0.00697 | 0.00146 | 0.00396 | 8.44E-04 |
| **Acetate** | Control | 0.08426 | 3.65E-05 | 0.08426 | 3.65E-05 |
|  | Low | 0.14089 | 0.00166 | 0.14089 | 0.00166 |
|  | High | 0.10147 | 0.00558 | 0.10147 | 0.00558 |
| **Butyrate** | Control | 0.17998 | 2.45E-04 | 0.14398 | 1.96E-04 |
|  | Low | 0.13661 | 0.00844 | 0.10929 | 0.00675 |
|  | High | 0.18238 | 0.01401 | 0.14194 | 0.0056 |
| **Acetone** | Control | 0.10243 | 0.00109 | 0.07683 | 8.16E-04 |
|  | Low | 0.06755 | 0.01407 | 0.05067 | 0.01056 |
|  | High | 0.10225 | 0.00863 | 0.07669 | 0.00647 |
| **Butanol** | Control | 0.10352 | 0.00854 | 0.06901 | 0.00562 |
|  | Low | 0.20534 | 0.00846 | 0.1369 | 0.00607 |
|  | High | 0.24265 | 0.0096 | 0.16176 | 0.00646 |

Table Supplemental 3.

Table 3. Experimental percentage of measured input synthesis gas. Inputs were balanced with N_2_, and corresponding values of its components as percentage of volume and millimols per hour. Flow: 12.48 L/h. Synthesis gas 100% contains 20% CO, 20% CO_2_ and 10% H_2_.

| Synthesis gas | CO | | CO_2_ | | H_2_ | |
| --- | --- | --- | --- | --- | --- | --- |
| % | % | mmol/h | % | mmol/h | % | mmol/h |
| 0 | 0 | 0 | 0 | 0 | 0 | 0 |
| 9 | 0.96 | 4.70 | 0.98 | 4.83 | 0.38 | 1.90 |
| 32 | 6.06 | 29.71 | 5.71 | 28.02 | 2.20 | 10.80 |
| 60 | 11.17 | 54.77 | 11.01 | 53.99 | 4.40 | 21.60 |

Table Supplemental 4.

Table 4. Carbon and carbon and energy balances of chemostat (D = 0.135 h^-1^) cultures of *Clostridium beijerinckii* SA-1 continuously sparged with different concentrations of synthesis gas (F = 12.48 L/h).

| **Gas Input** | Condition (Synthesis gas) | CO | CO_2_ | H_2_ |  |
| --- | --- | --- | --- | --- | --- |
|  |  | mmol/h | mmol/h | mmol/h |  |
|  | Control | 0 | 0 | 0 |  |
|  | Low | 4.70758 | 4.83924 | 1.9004 |  |
|  | Medium | 29.71662 | 28.02141 | 10.801 |  |
|  | High | 54.7747 | 53.9901 | 21.6077 |  |
| **Biomass** | Condition (Synthesis gas) | C and e balance | | C-balance | |
|  |  | Cmol/Cmol | SD | Cmol/Cmol | SD |
|  | Control | 0.27713 | 0.00454 | 0.26457 | 0.00433 |
|  | Low | 0.23768 | 0.02253 | 0.2269 | 0.02151 |
|  | Medium | 0.2609 | 0.02192 | 0.24906 | 0.02093 |
|  | High | 0.27314 | 0.04022 | 0.26076 | 0.03839 |
| **Ethanol** | Control | 0.00816 | 5.88E-04 | 0.00544 | 3.92E-04 |
|  | Low | 0.01186 | 0.00187 | 0.00791 | 0.00125 |
|  | Medium | 0.01271 | 0.0029 | 0.00847 | 0.00193 |
|  | High | 0.01333 | 0.00298 | 0.00888 | 0.00199 |
| **Acetate** | Control | 0.15653 | 0.03458 | 0.15653 | 0.03458 |
|  | Low | 0.16761 | 0.04969 | 0.16761 | 0.04969 |
|  | Medium | 0.19967 | 0.03305 | 0.19967 | 0.03305 |
|  | High | 0.19173 | 0.01012 | 0.19173 | 0.01012 |
| **Butyrate** | Control | 0.20984 | 0.09739 | 0.16788 | 0.07791 |
|  | Low | 0.25549 | 0.0776 | 0.20439 | 0.06208 |
|  | Medium | 0.30748 | 0.06021 | 0.24598 | 0.04817 |
|  | High | 0.37488 | 0.13626 | 0.2999 | 0.10901 |
| **Acetone** | Control | 0.06156 | 0.01076 | 0.04617 | 0.00807 |
|  | Low | 0.04005 | 0.00394 | 0.03004 | 0.00296 |
|  | Medium | 0.03977 | 0.00175 | 0.02983 | 0.00131 |
|  | High | 0.02488 | 0.00634 | 0.01866 | 0.00476 |
| **Butanol** | Control | 0.02312 | 0.01174 | 0.01541 | 0.00783 |
|  | Low | 0.05914 | 0.02468 | 0.03943 | 0.01646 |
|  | Medium | 0.08433 | 0.03469 | 0.05622 | 0.02312 |
|  | High | 0.11344 | 0.03872 | 0.07563 | 0.02581 |
